# Melanoma Inhibitory Activity (MIA) increases the invasiveness of pancreatic cancer cells

**DOI:** 10.1186/1475-2867-5-3

**Published:** 2005-02-14

**Authors:** Jamael El Fitori, Jörg Kleeff, Nathalia A Giese, Ahmed Guweidhi, Anja K Bosserhoff, Markus W Büchler, Helmut Friess

**Affiliations:** 1 Department of General Surgery, University of Heidelberg, Germany; 2 Institute of Pathology, University of Regensburg, Germany

**Keywords:** MIA, pancreatic cancer, invasion, metastasis, tumor cell invasion

## Abstract

**Background:**

Melanoma inhibitory activity (MIA) is a small secreted protein that interacts with extracellular matrix proteins. Its over-expression promotes the metastatic behavior of malignant melanoma, thus making it a potential prognostic marker in this disease. In the present study, the expression and functional role of MIA was analyzed in pancreatic cancer by quantitative real-time PCR (QRT-PCR), immunohistochemistry, immunoblot analysis and ELISA. To determine the effects of MIA on tumor cell growth and invasion, MTT cell growth assays and modified Boyden chamber invasion assays were used.

**Results:**

The mRNA expression of MIA was 42-fold increased in pancreatic cancers in comparison to normal pancreatic tissues (p < 0.01). In contrast, MIA serum levels were not significantly different between healthy donors and pancreatic cancer patients. In pancreatic tissues, MIA was predominantly localized in malignant cells and in tubular complexes of cancer specimens, whereas normal ductal cells, acinar cells and islets were devoid of MIA immunoreactivity. MIA significantly promoted the invasiveness of cultured pancreatic cancer cells without influencing cell proliferation.

**Conclusion:**

MIA is over-expressed in pancreatic cancer and has the potential of promoting the invasiveness of pancreatic cancer cells.

## Background

Despite improvements in diagnosis and treatment, pancreatic cancer remains one of the most common causes of cancer-related deaths in the world [1]. One of the reasons for the dismal prognosis is the propensity of pancreatic cancer cells to invade surrounding tissues and to metastasize. Melanoma inhibitory activity (MIA) is a small secreted protein normally expressed in cartilage and also produced by malignant melanoma cells and to a lesser degree by breast, colon cancer, and glioblastoma cells [2-4]. The exact biological functions of MIA are still unclear, but recent evidence indicates an important role of MIA in tumor progression and metastasis. MIA has been shown to interact with the components of the extracellular matrix, such as fibronectin and laminin, possibly via the binding motif for integrins. For example, MIA inhibits the attachment of suspended melanoma cells to surfaces coated with laminin or fibronectin [5]. In addition, overexpression of MIA in melanoma cells induces an aggressive tumor type by enhancing the metastatic potential. It has been shown that there is a correlation between increased plasma levels of MIA and a more advanced metastatic disease state in malignant melanoma patients [6]. Previously, using DNA array technology, we have demonstrated an increase of MIA mRNA expression in pancreatic cancer in comparison with the normal pancreas [7]. In the present study, we have further investigated the expression and localization of MIA, and its functional role in pancreatic cancer.

## Results

**Figure 1:**
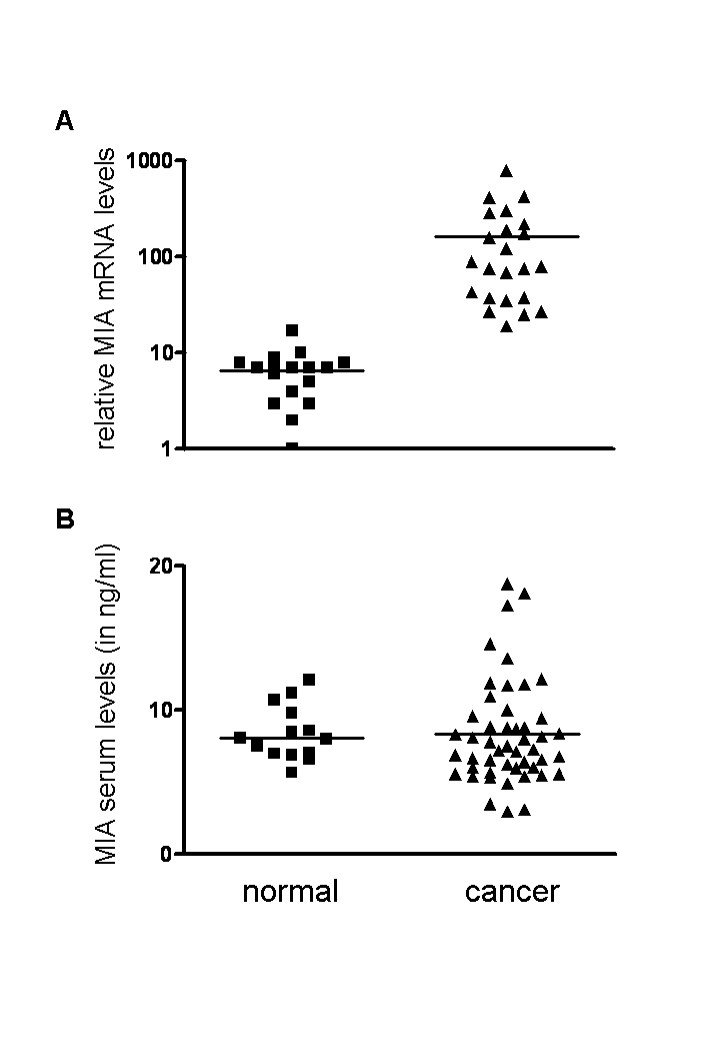
**MIA mRNA expression and serum levels. A: **MIA mRNA values in normal pancreatic tissues and pancreatic ductal adenocarcinoma (cancer) tissues by real-time quantitative polymerase chain reaction, as described in the *Methods *section. Values were normalized to housekeeping genes (cyclophilin B and hypoxanthine guanine phosphoribosyltransferase). **B: **ELISA was carried out as described in the *Methods *section. Fifty pancreatic cancer sera samples and 14 healthy donor sera samples were analyzed. The horizontal lines represent the mean expression levels.

**Figure 2:**
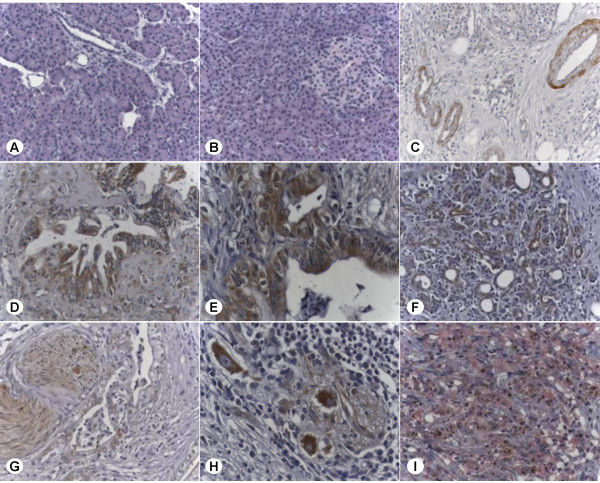
**Localization of MIA in pancreatic tissues. **MIA localization in pancreatic tissues: immunohistochemistry using a MIA specific antibody was carried out as described in the *Methods *section. **A-C: **normal pancreatic tissues; **D-H: **pancreatic cancers; **I: **malignant melanoma metastasis to the peritoneum. Note that in melanoma metastasis the signal is red (using HistoMark Red phosphatase system) to differentiate the staining from the brown pigment in melanoma cells.

To quantify the mRNA expression of MIA in pancreatic tissues, QRT-PCR was performed using RNA from pancreatic cancer tissues (n = 23) and normal pancreatic tissue samples (n = 17). The analysis revealed a 42 ± 28-fold increase (p = 0.0013) of MIA mRNA levels in pancreatic cancer tissues compared to the normal pancreas (Fig. 1A). To determine the exact localization of MIA in pancreatic tissues, immunostaining was carried out in 32 primary pancreatic cancers and in 17 normal pancreatic tissue samples. In the normal pancreas, MIA immunoreactivity was absent in ductal, acinar and islets cells, but was observed in the muscular layer of vessels (Fig. 2A–C). In contrast, in pancreatic cancer tissues, MIA immunoreactivity was moderate in the cancer cells and in tubular complexes in CP-like lesions adjacent to the tumor mass (Fig. 2D–F), and in blood vessels and nerve ganglia (Fig. 2G,H). Further analysis to correlate different tumor stages and grades with MIA immunoreactivity in pancreatic cancer tissues was performed. No difference between tumor stages, grades and MIA immunostaining was observed. To ensure the specificity of the antibody used, a malignant melanoma metastasis to the peritoneum was also analyzed. Strong cytoplasmic MIA staining of the malignant melanoma cells but not the surrounding tissues could be detected (Fig. 2I). Since a correlation between increased serum levels of MIA in malignant melanoma and more advanced metastatic disease has been reported previously, we next investigated MIA serum levels in pancreatic cancer patients and healthy donors. The mean values of MIA serum levels were 8.3 ± 3.56 ng/ml in pancreatic cancer patients (n = 50) and 8.82 ± 2.01 ng/ml in control subjects (n = 14) (n.s.) (Fig. 1B). Further analysis revealed that there was also no significant difference between patient groups with different tumor stages or grades.

**Figure 3:**
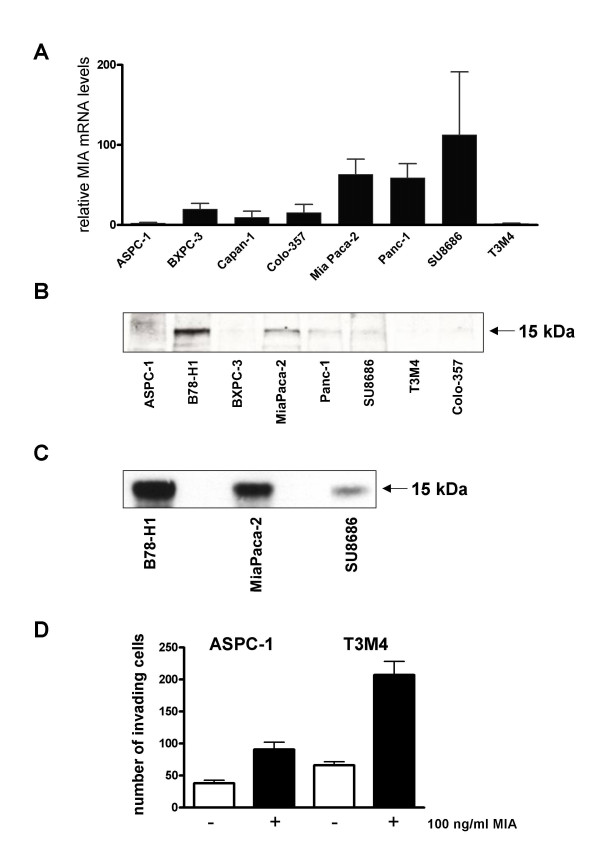
**Expression and effects of MIA in cultured pancreatic cancer cells. A: **MIA mRNA levels in indicated pancreatic cancer cell lines were determined by real-time quantitative polymerase chain reaction, as described in the *Methods *section. Data are presented as median + SD of MIA mRNA copies per μl of input cDNA normalized to housekeeping genes CPB and HPRT. **B: **30 μg protein lysates of the indicated cell lines were subjected to immunoblotting analysis using a specific MIA antibody, as described in the *Methods *section. **C: **Immunoprecipitation analysis was carried out as described in the *Methods *section. **D: **An in vitro cell invasion assay was performed using 8 μM filters coated with Matrigel, as described in the *Methods *section. 4 × 10^5^of the indicated pancreatic cancer cells were seeded onto the filters in 10% serum overnight, and then treated as indicated for 24 h. Invaded cells were stained and counted. The values shown are the mean ± SEM obtained from three independent experiments.

To determine the functional role of MIA in pancreatic cancer, we first investigated MIA expression in pancreatic cell lines. QRT-PCR analysis revealed relatively high MIA mRNA levels in Mia PaCa-2, Panc-1, and SU8686 pancreatic cancer cells compared to the other cell lines (Fig. 3A). Immunoblot analysis was employed to evaluate MIA protein levels in pancreatic cancer cell lines. This analysis revealed a band of 15 kDa corresponding to the known size of MIA in the control melanoma cell line B16 (B78/H1), whereas it was only weakly present in Mia Paca-2, Panc-1, and SU8686 pancreatic cancer cell lines, and below the level of detection in the other tested cell lines (Fig 3B). To confirm the specificity of the signal, immunoprecipitation was performed. This demonstrated an immunospecific band at 15 kDa in B78/H1, Mia PaCa-2, and SU8686 cell lines (Fig. 3C).

In order to determine the effects of MIA on the proliferation of pancreatic cancer cells, MTT cell growth assays were performed next. This analysis revealed no significant effects of MIA on the proliferation of pancreatic cancer cells (data not shown). To analyze whether MIA may promote invasiveness of pancreatic cancer cells, Matrigel-based invasion assays using a modified Boyden chamber were carried out in T3M4 and Aspc-1 pancreatic cancer cell lines that exhibited relatively low MIA expression levels according to QRT-PCR and Western blot data. Added to the top chamber as described in *Methods *section, MIA significantly increased the invasion of Aspc-1 by 2.4-fold (p < 0.001) and the invasion of T3M4 by 3.1-fold (p < 0.0001) (Fig. 3D). To investigate possible mechanisms of MIA overexpression in pancreatic cancer cells, we analyzed whether micro-environmental changes may modulate MIA expression. Neither TGF-β1 nor hypoxia was able to alter MIA mRNA expression, according to the QRT-PCR analysis of correspondingly treated pancreatic cancer cell lines (data not shown).

## Discussion

One of the most devastating aspects of malignant growth is the emergence of cancer foci in organs distant from the primary tumor, with most cancer mortality being related to metastases. Thus, understanding the molecular mechanisms underlying the metastatic process is one of the most important issues in cancer research.

Pancreatic cancer is characterized by aggressive local tumor growth and early systemic tumor spread [8-10]. Many factors are involved in transforming pancreatic cancer into a highly aggressive and metastatic disease, such as alterations in cell-cell interaction [11], deregulated expression of extracellular proteases [12], and metastasis-associated genes such as KAI-1, heparanase [13,14] and a number of other molecules.

Another important aspect of the metastasis process is neo-angiogenesis. Angiogenesis itself encompasses a cascade of sequential processes emanating from microvascular endothelial cells, which are stimulated to proliferate, degrade the endothelial basement membranes of parental vessels, migrate, penetrate host stroma, and initiate a capillary sprout [15]. Numerous angiogenic factors are overexpressed in pancreatic cancer, including vascular endothelial growth factor (VEGF), bFGF, and angiogenin, as well as members of the TGF-β, and FGF gene families [16-20]. In order to migrate and metastasize, cancer cells have to overcome and move through natural barriers created by cell-cell and cell-extracellular matrix (ECM) adhesion structures. Any destruction in cell-cell and ECM networks will facilitate motility and allow the cancer cells to migrate and metastasize. The invasion and metastatic potential of cancer cells depends on their intrinsic properties and the host microenvironment [21].

Melanoma inhibitory activity (MIA) increases cell motility by decreasing the attachment of the cells to the extracellular matrix (ECM). Overexpression of MIA leads to increased metastasis of malignant melanoma cells by enhancing invasion and extravasation [22,23]. In the present study we show by QRT-PCR and immunohistochemistry that MIA is significantly over-expressed in pancreatic cancer in comparison with normal pancreatic tissues. These data are in agreement with findings in malignant melanoma and breast cancer, in which MIA is also highly expressed [6,24,25].

In contrast to observations in malignant melanoma, where MIA has been established as a reliable marker for prognosis [6,24], we could not detect either a significant difference of MIA serum levels between pancreatic cancer patients and donors or a significant difference between patients at different stages of pancreatic cancer. Therefore, MIA cannot serve as a diagnostic or prognostic marker in pancreatic cancer.

The reason why high MIA mRNA levels lead to high serum levels in malignant melanoma, but not in pancreatic cancer, is currently not known. As to the possible role of MIA in pancreatic cancer pathogenesis, MIA had no effect on the proliferation of pancreatic cancer cells, similar to previous experiments employing fibroblasts, keratinocytes, endothelial cells and lymphocytes. The only cellular system in which MIA has been found to influence cell growth is melanoma cells; in these cells, MIA exerts anti-proliferative effects [26].

Although MIA did not affect the growth of pancreatic cancer cells in vitro, its impact on the invasion was striking. Interestingly, promotion of invasiveness of pancreatic cancer contrasts the previously demonstrated decrease in the invasion of MIA-treated malignant melanoma cells [26]. A possible explanation for this dissimilarity might be that MIA has no effect in detachment of pancreatic cancer cells from the ECM, like in malignant melanoma [26]. Alternatively, the status of MIA-interacting partners or downstream targets in the MIA signaling pathway may differ between pancreatic and melanoma cancer cells. So far, a possible correlation between MIA and other proteins known to be involved in pancreatic cancer metastasis – such as extracellular proteases, MMP, VEGF and bFGF – has not been studied. Therefore, the mechanism responsible for the invasion-promoting action of MIA requires further evaluation.

## Conclusion

In conclusion, our study shows a striking overexpression of MIA in cancerous pancreatic tissues without consequent elevation of MIA in the circulation. Involvement of MIA in regulation of invasiveness of pancreatic cancer cells indicates that this protein may serve as a novel therapeutic target in the search for anti-metastatic drugs.

## Methods

### Patients and tissues

Pancreatic cancer tissue samples were collected from 55 patients who underwent pancreatic cancer resection in the Department of General Surgery at the University of Heidelberg, Germany, and in the Department of Visceral and Transplantation Surgery at the University of Bern, Switzerland. Twelve cases were stage I, 13 cases stage II, 23 cases stage III, and 7 cases stage IV pancreatic adenocarcinomas, according to the Union International Contre Le Cancer (UICC) system. According to routine pathological grading, 16 cases were well-differentiated, 22 moderately differentiated, and 17 poorly differentiated. Normal pancreatic tissues were collected from 34 healthy organ donors. Pancreatic tissues were either frozen in liquid nitrogen and stored at -80°C (for RNA and protein extraction) or immediately fixed in 4% paraformaldehyde solution and subsequently embedded in paraffin. In order to determine MIA serum concentrations, sera from 50 pancreatic cancer patients (35 male, 15 female; median age 59 years; range 29–80 years) and healthy volunteers (14 male; median age 27 years; range 25–35 years) were collected at the Department of General Surgery, University of Heidelberg, Germany. Written informed consent was obtained from all patients. The study was approved by the Ethics Committees of the Universities of Bern, Switzerland, and Heidelberg, Germany.

### Cell lines and culture conditions

Mia PaCa-2, T3M4, Aspc-1, Bxpc-3, Capan-1, Colo-357, SU8686 and Panc-1 pancreatic cancer cells and B16 (cloneB78/H1) mouse melanoma cells were grown in RPMI 1640 medium containing 10% FBS (fetal bovine serum), 100 U/ml penicillin and 100 μg/ml streptomycin (Invitrogen, Karlsruhe, Germany). Cells were maintained in a 37°C humidified atmosphere saturated with 5% CO_2_. For TGF-β1 induction experiments, pancreatic cancer cells were seeded in 10 cm dishes in 10% FBS growth medium and allowed to attach for 12 hrs. Growth medium was replaced by serum-reduced medium (0.5% FBS), supplemented with 200 pM TGF-β1 for the indicated time periods. For experimental hypoxia, cells were subjected to a hypoxic microenvironment by one hour-long flushing in a special incubator chamber with an anoxic gas mixture (89.25% N_2_, 10% CO_2, _0.75%O_2_) and sealing of the unit.

### Real-time quantitative polymerase chain reaction (QRT-PCR)

All reagents and equipment for mRNA/cDNA preparation were purchased from Roche Applied Science (Mannheim, Germany). mRNA was prepared by automated isolation using MagNA Pure LC instrument and isolation kits I (for cells) and II (for tissue). cDNA was prepared using a 1st strand cDNA synthesis kit for RT-PCR according to the manufacturer's instructions. Real-time PCR was performed with the Light Cycler Fast Start DNA SYBR Green kit [27]. The number of specific transcripts was normalized to housekeeping genes (cyclophilin B and hypoxanthine guanine phosphoribosyltransferase, HPRT). All primers were obtained from Search-LC (Heidelberg, Germany).

### Immunohistochemistry

Briefly, consecutive paraffin-embedded tissue sections (5 μm thick) were deparaffinized and rehydrated. Antigen retrieval was performed by pretreatment of the slides in citrate buffer (pH 6.0) in a microwave oven for 10 min. Thereafter, slides were cooled to room temperature in deionized water for 5 min. After blocking of endogenous peroxidase activity with 0.3% hydrogen peroxide and washing in deionized water 3 times for 10 min, the sections were blocked for 1 h at room temperature with normal rabbit serum (DAKO, Hamburg, Germany), then incubated with primary goat polyclonal anti-MIA antibody (A-20, Santa Cruz Biotechnology, Santa Cruz, CA; dilution 1:35 in normal rabbit serum) overnight at 4°C. The slides were rinsed with washing buffer (Tris-buffered saline with 0.1% BSA) and incubated with secondary rabbit anti-goat HRPO-labeled IgG (Sigma-Aldrich, Taufkirchen, Germany), diluted 1:200 for 45 min at room temperature. After color reaction, tissues were counterstained with Mayer's hematoxylin. For negative control, appropriately diluted goat IgG was used instead of the primary antibody.

### Enzyme-linked immunosorbent assay (ELISA)

The amount of secreted MIA protein in cell culture supernatants and serum samples was determined using a one-step MIA ELISA (Roche Diagnostic GmbH, Mannheim, Germany) according to the manufacturer's instructions.

### Immunoblot

Cells were washed with ice-cold PBS and collected in lysis buffer (50 mM Tris-HCl, 100 mM NaCl, 2 mM EDTA, 1% SDS) containing the Complete mini-EDTA-free protease inhibitor cocktail tablets from Roche (Roche Applied Science, Mannheim, Germany). Lysates were centrifuged at 13,000 rpm at 4°C for 30 min, the supernatants were collected, and protein concentrations were measured with the BCA protein assay (Pierce Chemical Co., Rockford, IL, USA) using BSA as protein standard. 20 μg of protein were mixed with loading buffer, heated at 95°C for 5 min, separated on 12% SDS polyacrylamide gels, and transferred onto nitrocellulose membrane at 100 V for 90 min. Membranes were blocked in 5% non-fat milk in TBS-T (20 mM Tris-HCl, 150 mM NaCl, 0.1% Tween-20) for 1 h, incubated overnight at 4°C with anti-MIA antibody (A-20, Santa Cruz) and exposed to secondary HRPO-labeled donkey anti-goat antibody (Santa Cruz) for 1 h at room temperature. The signal detection was performed using the ECL system (Amersham Life Science, Amersham, UK).

### Immunoprecipitation

For immunoprecipitation, pancreatic cell lines (Mia PaCa-2 and SU8686) were suspended in lysis buffer (50 mM Tris, 150 mM NaCl, 1% Triton X-100, 25 mM NaF, 10% glycerol, 1 mM PMSF) supplemented with the Complete-TM mixture of proteinase inhibitors (Roche Diagnostic, Mannheim, Germany) and incubated for 30 min on ice. After centrifugation, the supernatant was transferred into a fresh vial, pre-cleared with protein A-Sepharose beads (Santa Cruz) and incubated with 50 μl anti-MIA antibody (A-20, Santa Cruz) overnight at 4°C. Following addition of 30 μl of protein A-Sepharose for 1 h at 4°C, the mixture was pelleted, washed three times with lysis buffer, and resuspended in Laemmli sample buffer.

### MTT cell growth assays

Cell growth experiments were performed using the 3-(4, 5-methylthiazol-2-yl)-2, 5-diphenyltetrazolium bromide (MTT) assay. Pancreatic cancer cells were seeded at a density of 5000 cells/well in 96-well plates, grown overnight, and then exposed to different concentrations of recombinant MIA protein as indicated. After 24 h, MTT was added (50 μg/well) for 4 hours. Formazan products were solubilized with acidic isopropanol, and the optical density was measured at 570 nm.

### Invasion assays

Invasion assays were performed in a BD Biocoat Matrigel Invasion Chamber with 8-μm pore size (BD Biosciences, Heidelberg, Germany) according to the manufacturer's instructions. The Matrigel was rehydrated with 500 μl DMEM (serum-free) and incubated in a 37°C, 5% CO2 atmosphere for 2 h. 5 × 10^4 ^cells were incubated for 24 h and subsequently treated with MIA (100 ng/ml) [26], which was added to the top chamber and incubated for 24 h. The non-invading cells were removed from the upper surface of the membrane with cotton-tipped swabs. Cells adhering to the lower surface were fixed with 75% methanol mixed with 25% acetone and stained with 1% toluidine blue (Sigma-Aldrich, Taufkirchen, Germany). The whole membrane was scanned using the software of the Zeiss KS300 and Zeiss AxioCam HR system (Jena, Germany). To calculate the total number of all invading cells, the cells were counted in every cut-out of the mosaic image of the whole membrane using the same software. The assays were performed in duplicate and repeated three times.

## Competing interests

The author(s) declare that they have no competing interests.

## Authors' contribution

JEF, NG, and AG carried out all the experiments, and participated in data analysis and interpretation. JK, MWB, and HF conceived of the study, and participated in its design and coordination. JK, NG, and AKB analyzed and interpreted the data and drafted the manuscript. All authors read and approved the final version.
